# Yeast thioredoxin reductase Trr1p controls TORC1-regulated processes

**DOI:** 10.1038/s41598-018-34908-4

**Published:** 2018-11-07

**Authors:** Cecilia Picazo, Emilia Matallana, Agustín Aranda

**Affiliations:** Institute for Integrative Systems Biology, I2SysBio, University of Valencia-CSIC, Valencia, Spain

## Abstract

The thioredoxin system plays a predominant role in the control of cellular redox status. Thioredoxin reductase fuels the system with reducing power in the form of NADPH. The TORC1 complex promotes growth and protein synthesis when nutrients, particularly amino acids, are abundant. It also represses catabolic processes, like autophagy, which are activated during starvation. We analyzed the impact of yeast cytosolic thioredoxin reductase *TRR1* deletion under different environmental conditions. It shortens chronological life span and reduces growth in grape juice fermentation. *TRR1* deletion has a global impact on metabolism during fermentation. As expected, it reduces oxidative stress tolerance, but a compensatory response is triggered, with catalase and glutathione increasing. Unexpectedly, *TRR1* deletion causes sensitivity to the inhibitors of the TORC1 pathway, such as rapamycin. This correlates with low Tor2p kinase levels and indicates a direct role of Trr1p in its stability. Markers of TORC1 activity, however, suggest increased TORC1 activity. The autophagy caused by nitrogen starvation is reduced in the *trr1*Δ mutant. Ribosomal protein Rsp6p is dephosphorylated in the presence of rapamycin. This dephosphorylation diminishes in the *TRR1* deletion strain. These results show a complex network of interactions between thioredoxin reductase Trr1p and the processes controlled by TOR.

## Introduction

In a single cell organism like the yeast *Saccharomyces cerevisiae*, metabolism and stress response are tightly intertwined^1^. When environmental conditions suddenly change, the fast rearrangement of metabolism and activation of the protection and defense mechanism are necessary to prevent cell viability loss. Nutrient-sensing pathways have been profoundly studied in budding yeast^2,3^. When nutrients are plenty, two main pathways contribute to cell growth, division and protein synthesis: Protein Kinase A (PKA) and the Target Of Rapamycin (TOR). These two main routes crosstalk, but PKA is activated basically by the presence of fermentable sugars, while TOR is fully activated when there is an easily assimilable nitrogen source, like ammonia or glutamine. The TOR pathway is articulated around the TORC1 complex^2,4^, which contains mainly Tor1p kinase, but Tor2p kinase can replace it, while TORC2 only contains Tor2p. TORC1 is inhibited by the drug rapamycin, anchored to the vacuolar/lysosomal membrane by the EGO complex, and its activity is regulated in an amino acid-dependent manner by Rag GTPases Gtr1/2p^5^. The active complex regulates many processes, promotes ribosome biosynthesis and translation through AGC kinase Sch9 activity, but prevents a general stress response (as explained below), and the use of non preferred nitrogen sources and autophagy. All these mechanisms are required to achieve full viability when nutrients are scarce and growth has ceased, a situation that cells face during chronological aging. It is known that inhibition by rapamycin and the mutation of TORC1 components extend life span^6^. Chronological life span is the time during which cells survive in the non dividing quiescent state, and TORC1 is the key pathway that orchestrates all these processes^4^. Although the way that TORC1 controls stress response has been well described, how stress conditions other that nutrient starvation regulate the TOR pathway is not fully known. For instance, as a protective mechanism under heat stress^7^, TORC1 is transiently sequestered into stress granules.

One of the main cellular stress responses takes place to deal with reactive oxygen species (ROS). The antioxidant machinery involves those enzymes devoted to detoxify these ROS, such as catalases (to deal with hydrogen peroxide) and superoxide dismutases, as well as systems to maintain cellular redox status^8,9^. There are two such systems; one is based on the tripeptide glutathione, while the other uses small proteins called thioredoxins. In the cytosol of *S. cerevisiae*, there are two thioredoxins, Trx1p and Trx2p, which restore the redox status of protein disulfide bonds that are essential for the functions of several enzymes, including ribonucleotide reductase. The system also includes a variety of peroxirredoxines, like Tsa1p, which detoxify peroxides^10^. The reductive power of the whole system comes from NADPH through the pivotal activity of thioredoxin reductase Trr1p, which restores the reduced status of both thioredoxins. As metabolic ROS are produced mainly by mitochondria during respiration, antioxidant machinery is very much required in post-diauxic states when it becomes essential to achieve a full life span^11^. *S. cerevisiae* is a biotechnologically relevant organism in food and biofuel industries where stress tolerance is a key factor for its performance^12^. In particular, oxidative stress tolerance is a determinant for yield and fermentative efficiency when yeast is produced as a dry starter^13^. It has been demonstrated that thioredoxin Trx2p overexpression improves both parameters by protecting key fermentative enzymes from oxidation^14^. Oxidative stress is also relevant for chronological aging during grape juice fermentation^15^, in combination with nutrient-sensing pathways, because the chemical inhibition of TORC1 also extends longevity under these conditions^16^. However, the balance of nutrients and the anaerobic state in grape juice fermentation influence the role of different pathway components^17^. Thioredoxin Trx2p has proven relevant for oxidative stress protection during biomass propagation^18^, and also regulates hexokinase 2^19^. Hxk2p is not only a glycolytic enzyme, but also a key player in glucose repression^20^.

In this work we studied a fundamental component of the cytosolic thioredoxin system, thioredoxin reductase Trr1p. We analyzed its role in longevity in different growth media and how it relates to other antioxidant proteins. We found a sensitivity of the *TRR1* deletion mutant to rapamycin that leads to the discovery of a new function of Trr1p in the regulation of the TORC1 activity and in the activation of autophagy. This function may be channeled through its interaction with EGO complex GTPase Gtr1p. Trr1p also controls the protein levels of TOR kinase Tor2p, which suggests a direct role in the amount or activity of the TORC1 complex.

## Results

### Trr1p influences growth and/or life span in a variety of growth media

**Figure 1:**
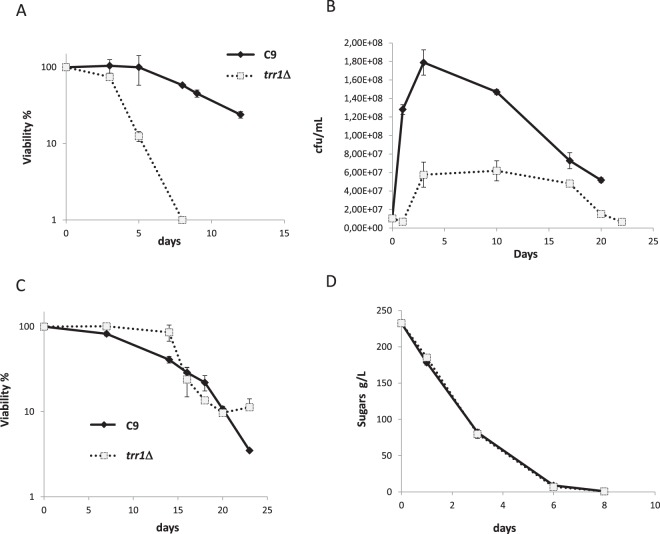
*TRR1* deletion impacts growth and longevity. (**A**) A standard chronological life span analysis in SC medium of wine yeast C9 and its *trr1*Δ derivative. (**B**) Viable cell number in the natural grape juice fermentations of the C9 wine strain and the *trr1*Δ deletion mutant. (**C**) Survival curve along the aforementioned vinifications. The viable cell number on day 3 from Panel (B) was taken as 100% survival. (**D**) Sugar consumption during grape juice fermentations described in panel (B). Experiments were done in triplicate. The mean and standard deviation are provided.

In order to study the impact of cytosolic thioredoxin reductase on chronological life span in a variety of environmental growth conditions, the *TRR1* gene was deleted in haploid wine yeast C9^21^ to allow us to also test the relevance of this gene on different growth conditions as laboratory *S. cerevisiae* strains do not perform well under grape juice fermentation^22^. The impact of *TRR1* deletion on chronological life span was tested in standard synthetic complete SC medium after 3 days of growth (Fig. 1A) when cells had ceased to divide, completely consumed carbon sources and entered the stationary phase. *TRR1* deletion caused a dramatic drop in viability. Therefore under these respiratory conditions, the thioredoxin system is necessary for longevity, which also occurs for laboratory strains^23^.

The performance of the *trr1*Δ mutant in natural grape juice fermentation was tested. The mutant displayed a growth defect when tested with an extended lag phase and lower cell density (Fig. 1B). By taking the highest point of cell viability as 100% survival, the CLS plot in grape juice fermentation was obtained (Fig. 1C), where no dramatic change in life span was observed. Therefore, the thioredoxin system does not seem necessary for longevity under this specific fermentative condition. The sugar consumption profile during grape juice fermentation showed no delay (Fig. 1D) despite the lower cell density reached by the *trr1*Δ mutant, which suggests that mutant cells would have stimulated sugar metabolism.

### Lack of thioredoxin reductase triggers compensatory antioxidant mechanisms

**Figure 2:**
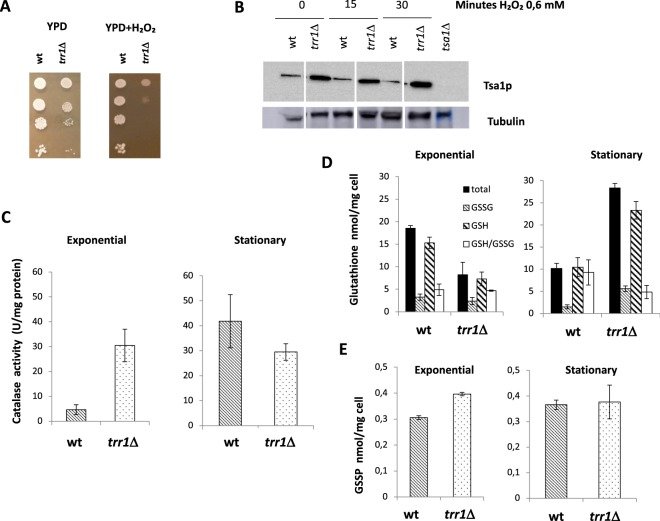
Trr1p plays a complex role in redox homeostasis. (**A**) Spot assays of the wine yeast C9 mutants and the *trr1*Δ mutant on YPD plates containing 3 mM H_2_O_2_. (**B**) Western blot against peroxiredoxin of both strains under hydrogen peroxide stress. Tubulin was used as a loading control. The picture was cropped to remove an unrelated mutant, marked with gaps (Supplementary Fig. S2). (**C**) Catalase in the exponential and stationary phase of growth in rich YPD medium in the wild type and mutant strain. (**D**) Quantification of reduced, oxidized and total glutathione under the same conditions as panel. (**C**,**E**) Protein-bound glutathione under the same conditions. Experiments were done in triplicate. The mean and standard deviation are provided.

The role of Trr1p in oxidative stress and its relationship with other oxidative stress proteins and pathways were analyzed. As expected, the mutant strain showed increased sensitivity to the oxidative insult caused by hydrogen peroxide (Fig. 2A). Due to the complex network of antioxidant mechanisms and the existence of compensatory effects, the accumulation of the main peroxiredoxin Tsa1p was analyzed in the *trr1*Δ mutant in complete minimal medium. As seen in Fig. 2B, an amount of peroxirredoxin increased even in the absence of oxidative stress, a finding that agrees with previous reports for lab strains^24^. In a similar way, catalase activity increased in the *trr1*Δ mutant when cells were grown exponentially (Fig. 2C). When cells reached the stationary phase, catalase activity increased as expected^25^, but under this condition, *TRR1* deletion did not further increase. Therefore, the *trr1*Δ mutant seems to contain a defect in the usual repression of the stress response during exponential growth in rich medium.

Due to the connection among different antioxidant cellular tools, and given the deficiency in the thioredoxin system, glutathione may become more relevant to compensate redox unbalances. To test this hypothesis, the amount of both reduced and oxidized glutathione was analyzed in the *trr1*Δ mutant (Fig. 2D). Glutathione metabolism is affected in the mutant as the total amount diminishes under exponential growth, but increases in the stationary phase. This may indicate that during respiratory metabolism, lack of *TRR1* leads to the synthesis of more glutathione. The GSH/GSSG ratio indicates that there is small difference in the redox status of cells, with only a slight decrease in the stationary phase, which indicates that oxidative damage may thus accumulate. As thioredoxins have been proposed to play a role in protein deglutathionylation^26^, the glutathione linked to proteins was analyzed (Fig. 2E). The *trr1*Δ mutant presents a larger amount of glutathione conjugated to protein during exponential growth, but not in the stationary phase when oxidative stress is higher.

### *TRR1* deletion impacts amino acid metabolism

**Figure 3:**
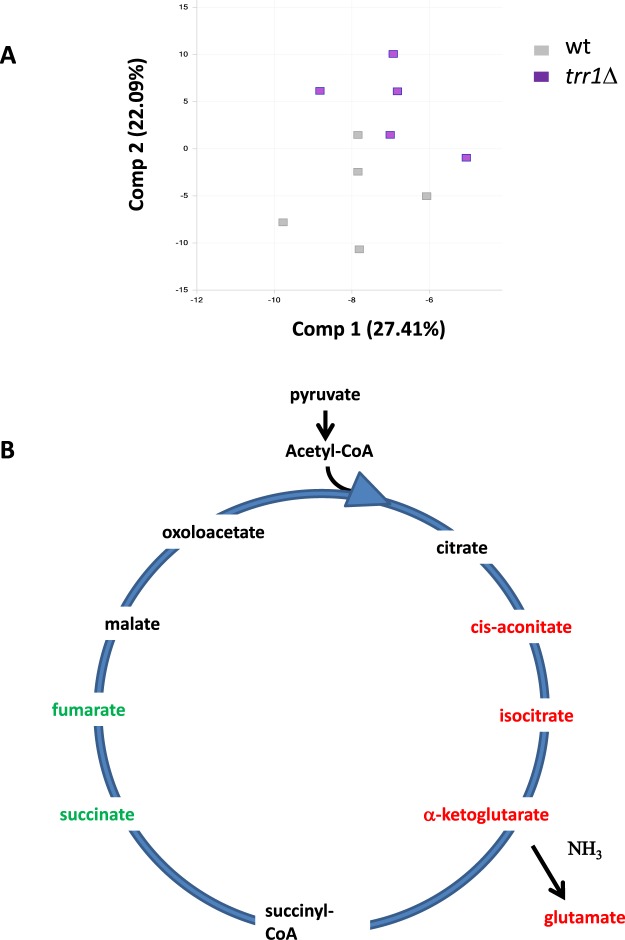
The *TRR1* deletion causes a global metabolomics alteration. (**A**) The PCA analysis of the metabolomic data of strains C9 and the *trr1*Δ mutant during wine making. Five replicas of each strain were tested. Each principal component is a linear combination of every metabolite, and principal components are uncorrelated. The number of principal components equals the number of observations. (**B**) The TCA pathway and its connection to glutamate synthesis. The metabolites up-regulated in *trr1*Δ are highlighted in red and the down-regulated metabolites are depicted in green.

*TRR1* deletion impacts growth during grape juice fermentation without affecting the sugar consumption profile. So we performed a comparative metabolomic analysis of the *trr1*Δ mutant and the C9 strain in synthetic must fermentation to gain more insight into the role of Trr1p in metabolism. Synthetic must is a controlled rich medium that allows our data to be compared to other global transcriptomic and metabolomic studies^27,28^. Cell samples were taken in the stationary phase, but with the sugar concentration still around 50 g/L so that cells did no divide, but remained metabolically active. Of the 423 identified compounds, 221 changed when comparing the *trr*1Δ mutant to the wild type, 160 increased in the mutant and 61 decreased (Supplemental Table S1). The PCA component analysis showed that the *trr1*Δ deletion had a broad impact on metabolism, but not dramatically (Fig. 3A). Under these conditions, 13 proteinogenic amino acids were slightly elevated in the *trr*1Δ mutant compared to the wild type. Only proline significantly reduced, while the other six did not significantly change. Furthermore, the modified amino acids, such as acetylated amino acids, were also higher. As these amino acid modifications occur post-translationally, their presence implies increased proteolysis, which suggests that this might be the cause of the higher levels of the amino acids observed in the *trr1*Δ mutant. A higher content of both oxidized and reduced glutathione, as seen under the laboratory conditions (Fig. 2D), suggests the up-regulation of glutathione synthesis as a compensatory mechanism. Elevated levels of γ-glutamyl-amino acids also reinforce the idea. Hydroxy-fatty acids are markers of oxidative stress, and some increased in the *trr1*Δ mutant strain, while others remained unchanged and some diminished. Therefore, the *trr1*Δ mutation does not seem to impose strong oxidative stress under this growth condition.

Regarding energy metabolism, increased intracellular glucose did not lead to higher pyruvate or acetyl-CoA, so the glycolytic flux did not seem to be up-regulated. The TCA metabolites that linked glycolysis to glutamate production, such as isocitrate and α-ketoglutarate, increased, while some intermediates on the other side of the cycle decreased, such as succinate and fumarate, (Fig. 3B). As glutamate dehydrogenase uses α-ketoglutarate in a key step to introduce ammonium into the metabolism, the effect of Trr1p is interesting and may explain the increase in other amino acids by transamination from glutamate. As the components of the NAD salvage pathways, including both NAD^+^ and NADH, increased, redox unbalance would not seem to be the cause.

### Trr1p is linked to the TORC1 pathway

**Figure 4:**
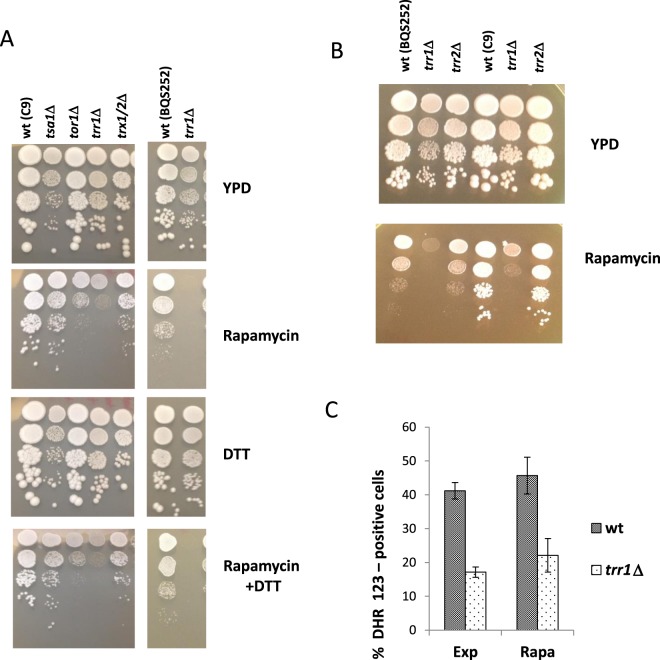
Trr1p is required for tolerance to rapamycin. (**A**) The spot analysis of sensitivity to rapamycin of mutants *tor1*Δ*, tsa1*Δ, *trx1*Δ*trx2*Δ and *trr1*Δ in the C9 strain. The YPD plates that contained 100 nM rapamycin, 1 mM DTT, or both, were used. Strain BQS252 and its *trr1*Δ mutant were also included. (**B**) A spot analysis of rapamycin sensitivity of the *trr2*Δ mutant in both genetic backgrounds. (**C**) Intracellular ROS in BQS252 and its *trr1*Δ mutant during exponential growth with or without rapamycin.

**Figure 5:**
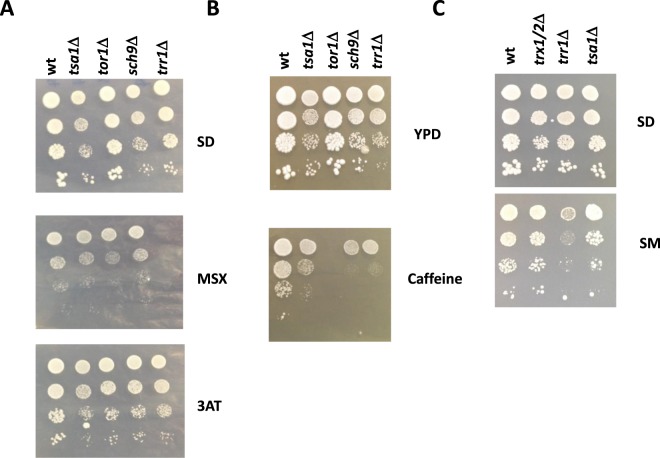
Sensitivity to other inhibitors that cause amino acid starvation. (**A**) The spot analysis in the minimal medium SD that contained methionine sulfoximine (MSX,100 mM) and aminotriazole (3AT, 10 mMM) of strain C9 and the mutants in genes *TRR1*, *TSA1*, *SCH9* and *TOR1*. (**B**) The spot analysis of tolerance to 10 mM caffeine on the rich medium YPD of the aforementioned mutants. (**C**) A similar experiment on the SD plates that contained 1 g/ml of sulfometuron methyl (SM).

As *TRR1* deletion impacted growth and metabolism, its connection to nutrient-sensing pathways was studied by using the inhibition of such pathways or specific metabolic steps (Figs 4 and 5). As we studied the relevance of TORC1 during winemaking by different approaches, we tested the effect of rapamycin inhibition on the *trr1*Δ mutant (Fig. 4A). Indeed the *trr1*Δ mutant was affected by rapamycin, showing hypersensitivity to it, which suggests that TORC1 has diminished activity in the mutant. This phenotype is not apparently linked to the activity of the thioredoxin system as neither the single mutants in cytosolic thioredoxins *TRX1* and *TRX2* (data not shown), nor the double mutant *trx*1Δ*trx2*Δ, displayed increased rapamycin sensitivity. Nor was the main target of thioredoxins, cytosolic peroxiredoxin Tsa1p, involved in the process. The mutant in TOR kinase *TOR1* was included as a positive control. The connection of Trr1p with the TOR pathway would not, therefore, seem to be related to its function in the thioredoxin system.

We analyzed the rapamycin sensitivity phenotype of the *trr1*Δ mutant in laboratory strain BQS252 (Fig. 4A). This stain is a haploid derivative of diploid strain FY1679, and is isogenic to strain S288c that contains only the *ura3–52* auxotrophy and no other mutations in amino acid biosynthetic genes that may alter the outcome when studying nitrogen-sensitive pathways. The mutation also causes rapamycin sensitivity, so the phenotype is not dependent on the genetic background. We also constructed the *TRR2* deletion, which codes for the only mitochondrial tiorredoxin reductase in *S. cerevisiae*, and we analyzed sensitivity to rapamycin and found no effect on the *trr2* mutant (Fig. 4B). So only Trr1p, and neither thioredoxins nor Trr2p, showed the connection to TOR pathway activity. To completely rule out the global redox status in the *trr1*Δ mutant causing the rapamycin sensitivity phenotype, we used antioxidant dithiotreitol (DTT) to check if it suppressed it after testing that DTT itself had no impact on growth (Fig. 4A). Rapamycin specifically inhibits mutants *tor1*Δ and *trr1*Δ, and this phenotype is not rescued by the combination of DTT and rapamycin. The same happens when the laboratory strain is used. Finally, cellular ROS were analyzed by cytometry in the wild type and the *trr1*Δ mutant laboratory strains during exponential growth in both the presence and absence of rapamycin (Fig. 4C). Rapamycin did not increase the ROS level in any strain, so TORC1 inhibition itself did not trigger oxidative damage. Interestingly, *TRR1* deletion lowered the ROS level during exponential growth, probably due to the activation of the above-described compensatory mechanism, such as catalase and a higher Tsa1p level.

Other metabolic inhibitors were tested to profoundly characterize the phenotype of the *TRR1* deletion (Fig. 5). Methionine sulfoximine (MSX) inhibits glutamine synthetase by causing starvation that inhibits TORC1^29^. Using minimal SD medium to score amino acid starvation, the *trr1*Δ mutant was also hypersensitive to MSX (Fig. 5A), more than *tor1*Δ and its downstream kinase *sch9*Δ mutants. Caffeine is believed to directly inhibit the TORC1 complex^30^, but also affects other pathways. The *trr1*Δ mutant showed increased sensitivity to caffeine (Fig. 5B), similarly to *sch9*Δ, but to a lesser extent than the *tor1*Δ mutant. These findings indicate that Trr1 indirectly affects caffeine sensitivity. 3-aminotriazole (3AT) is an inhibitor of histidine biosynthesis, causes histidine starvation and triggers the activation of General Amino Acid Control kinase (GAAC) Gcn2p. However, neither *trr1*Δ mutant nor any of the other tested mutants showed hypersensitivity to 3AT (Fig. 5A), which indicates that Trr1p is not involved in GAAC^31^, and that the effect of its mutation is not caused by global amino acid starvation. Another inhibitor of amino acid biosynthesis is sulfometuron methyl (SM), which prevents the synthesis of the branched amino acid family^32^. It also triggers GAAC, but leucine is also able to activate TORC1^33^. In this case, the *trr1*Δ mutant showed sensitivity to this inhibitor (Fig. 5C), which suggests a role in the response to leucine starvation. Therefore, cytosolic thiorredoxin reductase Trr1p might regulate specific amino acid biosynthetic pathways, likely through the modulation of TORC1 activity in a Gcn2-independent way.

### Molecular effects of *TRR1* deletion

**Figure 6:**
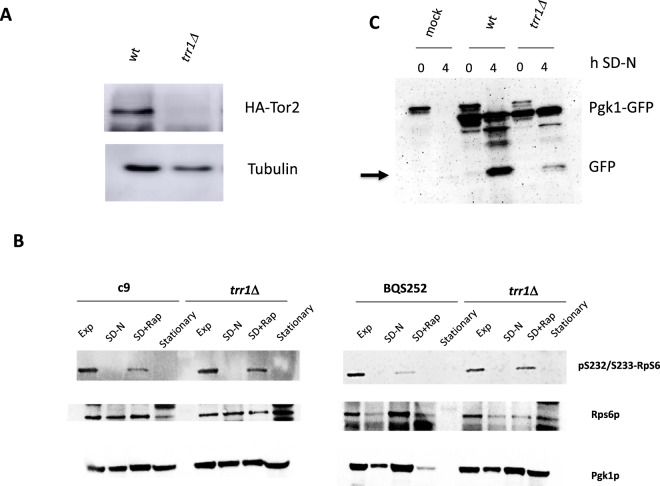
The *trr1*Δ mutant has altered Tor2 levels and TORC1 activity. (**A**) Western blot analysis against a HA-tagged version of *TOR2* in the BQS252 and its *trr1*Δ mutant. Cells were grown for 4 hours in galactose to express the construct. An antibody against HA tag was used to detect the protein level. Tubulin was used as a loading control. (**B**) Western blot against the phosphorylated isoform of ribosomal protein Rps6. The *TRR1* deletions in both wine yeast strain C9 and laboratory strain BQS252 were tested against rapamycin treatment, nitrogen starvation and in the stationary phase. Total Rps6 and Pgk1 were used as loading controls. (**C**) The autophagy analysis under nitrogen starvation of the Pgk1-GFP fusion of the *trr1*Δ mutant. The arrow indicates the GFP fragment that marks autophagy.

The strong sensitivity to rapamycin in the *trr1*Δ mutant suggests an altered TORC1 pathway. In their COOH-terminal FATC domain, both Target of Rapamycin kinases Tor1p and Tor2p possess two conserved cysteines to form a disulfide bond that stabilizes the protein^34^. The redox potential of such a FATC domain comes close to that of GSH/GSSG, and are more oxidizing than the thioredoxin system. Hence Trr1p should have enough reducing power to directly or indirectly break this disulfide bond and to then destabilize these kinases. To measure the amount of TOR kinase, we transformed the wild-type BQS252 strain and its *trr1*Δ mutant with an N-terminal HA tagged *TOR2* gene^35^. The cells grown exponentially on galactose to induce the expression of the tagged protein were used to detect Tor2p levels by Western blot (Fig. 6A). *TRR1* deletion led the Tor2p levels to significantly drop, which would explain its sensitivity to TORC1 inhibitors such as rapamycin.

To address the influence of thioredoxin reductase Trr1p on events under the control of TOR kinases, we analyzed rapamycin sensitivity at the molecular level. Ribosomal protein Rps6p is phosphorylated by AGC kinase Ypk3p in a TORC1-controlled process, so Rsp6p is dephosphorylated when rapamycin inhibits this kinase^36^. We analyzed the phosphorylation status of Rps6p under different conditions (Fig. 6B). In both the C9 and BQS252 genetic backgrounds, Rps6p was phosphorylated when cells were exponentially grown. A chemical inhibition of TORC1 by rapamycin resulted in lower phosphorylated Rps6p levels in both genetic backgrounds. *TRR1* deletion kept Rps6p phosphorylation elevated after addition of rapamycin in both backgrounds, which indicates lower sensitivity to rapamycin in the mutant and an alteration in the TORC1 complex. Complete Rps6p dephosphorylation was observed in the stationary phase, and also in the nitrogen starvation for both strains and mutants. These conditions are more complex and many pathways are involved, which suggests that Trr1p plays a TORC1-dependent role in Rps6p status.

Autophagy is a well-studied TORC1-controlled process. TORC1 inhibition by nutrient starvation leads to its activation to recycle cell components and to extend chronological aging. Different proteins can be used as autophagy markers, such as glycolytic enzyme Pgk1p, which in a fused version to GFP and allows the process to be followed by detecting GFP. Pgk1p-GFP autophagy was analyzed after 4 h under nitrogen starvation in SD medium (Fig. 6C), where the wild-type strain showed the GFP peptide that marked autophagy. In the *trr1*Δ mutant, the amount of GFP peptide was significantly lower and most of the fusion protein remained intact. Therefore, the *trr1*Δ mutant has a block of autophagy. The induction of autophagy by rapamycin is less dramatic that the one caused by nitrogen depletion and again it is lower in the *trr1*Δ mutant strain (Supplementary Fig. S1). Thus, our results with the *TRR1* deletion mutant point out the increased activity of TORC1 and/or lower sensitivity to nutrient signaling.

### *TRR1* genetically interacts with TORC1 GTPase *GTR1*

**Figure 7:**
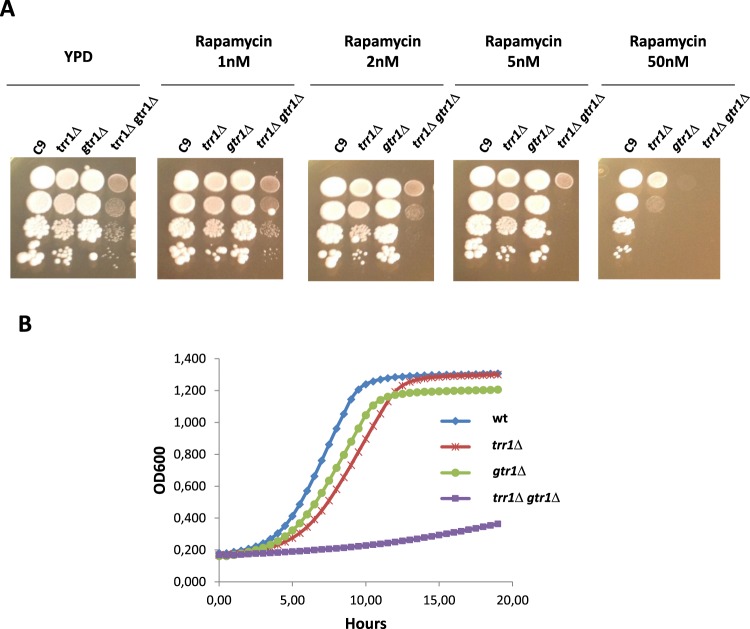
*TRR1* and the GTPase *GTR1* interact genetically. (**A**) The spot analysis of different amounts of rapamycin of the single *trr1*Δ and *gtr1*Δ mutants, and the double *trr1*Δ *gtr1*Δ mutant in strain C9. (**B**) Growth curves in the YPD rich medium of the strains used in section (**A**) performed by a Varioskan Lux plate reader at 30 °C with shaking.

In order to find new players to help explain the connection between Trr1p and the TORC1 pathway, databases were analyzed to search for interacting proteins. Trr1p was pulled down by a TAP-tagged version of GTPase Gtr1p^33^. Thus the *GTR1* gene was deleted and combined with the *TRR1* deletion in the C9 strain, and the single and double mutants were tested for their rapamycin sensitivity at different inhibitor concentrations (Fig. 7A). Around 100–200 nM is the usual concentration to test rapamycin sensitivity for wild-type strains. Both single mutant strains were already very sensitive to 50 nM rapamycin, and the *gtr1* mutant was even more sensitive. When the amount of rapamycin lowered to 2–5 nM, the single mutants showed a slight growth defect, but the double mutant became more sensitive. This scenario indicates a genetic interaction between both genes, and both proteins acted during the same process in relation to the TORC1 control. The same mutants were also tested in a different genetic background, strain BQS252 and the genetic interaction was confirmed (data not shown). When rapamycin was low, the *GTR1* deletion caused hypersensitivity to rapamycin, but the double mutant *trr1*Δ *gtr1*Δ increased even further, which confirmed this genetic interaction.

We found a growth defect in rich medium when the double mutant was constructed. To confirm this, a growth curve in YPD was obtained from the *trr1*Δ, *gtr1*Δ and *trr1*Δ *gtr1*Δ mutants in the C9 stain (Fig. 7B). The *trr1*Δ mutant showed a longer lag phase, may due to a lower amount of TORC1 and/or TORC2 complexes, but reached an equal final cell density as the parental strain, while the *gtr1*Δ mutant grew faster than the *trr1*Δ mutant, but reached lower cell density. The double mutant displayed a dramatic defect on growth in YPD, which suggests that the genetic interaction between *GTR1* and *TRR1* is essential for exponential growth, which is when TORC1 must be active.

## Discussion

Thioredoxin reductase Trr1p is a key component of the thioredoxin system as it provides reducing power to feed thioredoxins and peroxiredoxins so they can perform their antioxidant duties^8,9^. As expected, its deletion caused a defect in oxidative stress (Fig. 2A) and in other phenotypes, which matched its lesser antioxidant power as its decreased chronological life span (Fig. 1A). These are typical defects produced by mutations in antioxidant enzymes^37,38^ as metabolism in the stationary phase is respiratory and ROS production increases. The oxidative stress response is complex and many antioxidant systems cooperate to deal with this insult. Lack of Trr1p triggers compensatory mechanisms, e.g., an increase in catalase activity in the exponential phase (Fig. 2C), and in total and reduced glutathione in the stationary phase (Fig. 2D). *TRR1* deletion has been described to increase the levels of thioredoxins by activating antioxidant transcription factor Yap1p^39^. We tested whether the thioredoxin peroxidase Tsa1 levels also increased (Fig. 2A). Yap1p activation could be the molecular mechanism for the described responses. These compensatory mechanisms would explain the lower ROS levels in the *trr1*Δ mutant in the exponential phase (Fig. 4C). According to the free radical theory of aging^40^, this may seem inconsistent with the shortened life span. However, it has been previously taken into account that the impact of oxidants on aging is complex, as is the coordinated action of the different defenses and outputs; e.g., inhibition of catalases extends chronological life span by increasing H_2_O_2_, but reduces superoxide ions^41^. Such a hormetic effect may take place in the *trr1*Δ mutant or, alternatively, Trr1p might modulate processes other that the detoxification of peroxides, which also affect longevity.

Deletion of *TRR1* had a huge impact on growth during grape juice fermentation (Fig. 1B), which means that it played additional roles under other stressful conditions. Perhaps ROS production during the wine fermentative process is expected to be lower than in respiratory medium. In addition to its impact on growth, *TRR1* deletion alters metabolism on a global scale (Fig. 3). It increases the levels of most proteinogenic amino acids and alters TCA to increase glutamate levels. No metabolomic evidence exists for strong oxidative damage, so this metabolic alteration seems a direct effect of lack of Trr1p rather than a response to redox unbalance. Unfortunately, the proteomic analysis did not detect the reductive power donor for Trr1p, NADPH (probably due to its instability), but the rise in NADH indicated that it was not a question of there being a more oxidative cellular state in the *trr1*Δ mutant. It was not possible to compare our metabolomics data to other global analyses as the *TRR1* deletion was considered inviable in the global deletion project^42^, and its knock-out is not included in most used deletion strain collections. Our deletions were performed in two prototrophic strains for amino acid biosynthetic genes, which may suggest a potential interaction with the metabolic genes used as selective transformation markers in standard reference strains.

The alteration in metabolism caused by the *TRR1* deletion could be caused by effects on the TORC1 pathway, a central regulator of metabolism and growth. The amount of Tor2p protein lowered in the *trr1*Δ mutant (Fig. 6A). This could indicate a global reduction in the Tor1/2 proteins that would lead to a smaller amount of the TORC1 complex, and would explain the increased sensitivity to the TORC1 inhibitor rapamycin (Fig. 4A). That may lead as well to a lower TORC2 complex concentration, and the decrease of both complexes may explain the defect in growth observed for the *TRR1* deletion mutant in grape juice fermentation (Fig. 1B) and in rich YPD medium (Fig. 7B). The fact that growth is not greatly impaired may indicate that Tor2p is present in excess during these conditions in the wild type and a reduction in Tor2p may still provide enough TORC2 complex to promote a fair amount of growth in the *trr1*Δ mutant. The mutant also showed high sensitivity to the other chemicals that inhibited TORC1, such as caffeine and MSX, and not to 3AT which activated GAAC. Therefore, it is not a general alteration of amino acid metabolism. The alteration in the Tor kinase level could be direct or indirect. The formation of a disulfide bond in the C-terminal FATC domain of Tor1/2 kinases has been proposed to help the interaction of these proteins with the vacuolar membrane by increasing their levels^34^. Trr1p has the redox potential to directly or indirectly reduce this disulfide bond so it can play a role in stabilizing TOR kinases. However, if this were the case, a higher level of Tor2p would be expected in the *trr1*Δ mutant. We cannot rule out the role of Trr1p in a dynamic process during TORC1 assembly, which could lead to the premature degradation of TOR kinases that would explain these results.

The phosphorylation level of ribosomal protein Rsp6p depends on the activity of Ypk3p kinase^36,43^, which is activated by the TORC1 complex. The normal Rsp6p phosphorylation level in the *trr1*Δ mutant (Fig. 6B) suggests a compensatory mechanism, that may involve phosphatase regulation. However, the dephosphorylation triggered by TORC1 inhibition by rapamycin was blocked in the *TRR1* deletion strain (Fig. 6B), which was not the case when dephosporylation was caused by either nitrogen starvation or entry in the stationary phase. This scenario indicates that Trr1p targets specifically TORC1 activity, and its absence causes rapamycin insensitivity. In the Gonzalez *et al*.^36^ paper that first describe the Rps6p phosphorylation analysis, a rapamycin resistant *TOR1-1 TOR2-1* mutant show a similar output, high levels of phosphorylated Rps6p in the presence of rapamycin. That indicates that TORC1 complex in the *trr1*Δ mutant may be irresponsive to rapamycin in terms of Ypk3p activation, but it may have additional effects that impair growth in the long term. However, the autophagy triggered by nitrogen starvation is Trr1p-dependent (Fig. 6C) so different processes downstream of TORC1 may be affected by distinct stimuli. TORC2 has a positive role in autophagy during amino acid starvation^44^, while most experiments show an inhibitory effect of TORC1^45^. The fact that Trr1p may be affecting both complexes would explain contradictory results regarding TOR targets.

The possibility of a protein using NADPH as a cofactor to regulate TORC1 offers a novel way of regulation for this pathway in relation to cell redox status. The superoxide ion has been described to modify TORC1 sensitivity to rapamycin^46^ and the increase in ROS produced by the mitochondria reduced stress signaling via TORC1^47^. The fact that deletions of other thioredoxin system members, or of the other thioredoxin reductase, does not alter rapamycin sensitivity (Fig. 4), but suggests that TORC1 modulation by Trr1p should be specific, and not a general alteration of the redox status caused by the mutation of the thioredoxin system. The only known interaction between Trr1p and the TORC1 pathway is the physical interaction described between Trr1p and TORC1 regulatory GTPase Gtr1p^33^. We describe how these genes also interacted at the genetic level by causing dramatic sensitivity to rapamycin (Fig. 7). This additive effect of both mutations indicates that both proteins affect TORC1 by a different mechanism, although Trr1p may rely on its interaction with Gtr1p to be tethered to the TORC1 complex. Further studies on the function of Trr1p on TORC1 are required to obtain a complete picture of the potential regulatory mechanisms that work through this interaction.

## Methods

### Yeast strains and growth media

The yeast strains used in this work derive from haploid wine yeast C9^21^, and from laboratory strain BQS254 (*MAT*a *ura3-52*), which is a haploid strain that derives from FY1679^48^. To perform gene disruptions, recyclable selection marker *loxP*-*kanMX*-*loxP* from plasmid pUG6^49^ was used. The GFP tagging of *PGK1* was done with plasmid pFA6-GFP-KanMX6, designed by Longtine^50^. In order to construct the double mutant *trr1*Δ *gtr1*Δ, *kan*MX marker from *trr1*::*kan*MX was excised with *cre* recombinase using plasmid YEp351-cre-cyh^51^. HA-TOR2 plasmid pSEYC68-HA-TOR2 (pAS21) was a gift from Michael Hall (Addgene plasmid # 39207). Yeast transformations were performed by the lithium acetate method^52^.

Yeasts were usually grown in rich YPD medium (1% yeast extract, 2% bactopeptone, 2% glucose). The solid media contained 2% agar, and 20 μg/ml geneticin, whenever required. Complete minimal SC medium, which contained 0.17% yeast nitrogen base, 0.5% ammonium sulfate, 2% glucose and 0.2% drop-out mix with all the amino acids^53^, was used for the chronological life span experiments. SD is equal to SC without the mix of amino acids. Red grape juice (Bobal variety) was a gift from Bodegas Murviedro (Requena, Spain). It was sterilized overnight with 500 μg/l of dimethyl dicarbonate. Synthetic grape juice MS300 was made as previously described^54,55^.

### Chronological life span experiments and grape juice fermentation

The CLS experiments were carried out in the standard way in synthetic complete medium SC^56^. Precultures were grown overnight on YPD and inoculated in SC media at an OD_600_ of 0.1. After 3 days of growth at 30 °C, aliquots were taken, diluted and plated. Colonies were counted and the percentage of survival was calculated by taking day 3 of growth as 100% survival. Growth curves were performed by a Varioskan Lux plate reader.

The fermentation experiments done with the natural and synthetic grape juices were inoculated with the cells from the stationary cultures in YPD at a final concentration of 10^6^ cells/ml in filled-in conical centrifuge tubes with 30 ml of grape juice. Incubation was done by low shaking at 25 °C. Vinification progress was followed by determining colony-forming units (by serial dilution, plating and colony counting) and sugar consumption by the reaction to DNS (dinitro-3,5-salycilic acid)^16,57^.

### Redox parameters

ROS were measured by flow cytometry. Cells were washed twice and resuspended in 10 mM PBS (pH 7.2). Then dihydrorhodamine 123 (DHR 123, Sigma) was added at 5 μg/ml and cells were incubated in the dark for 90 min at 30 °C. Finally, cells were harvested, washed resuspended in PBS buffer and analyzed using the “Annexin V and Cell Death” channel of a flow cytometer Muse Cell Analyzer (Millipore). To measure catalase activity, extracts were obtained from 50 mg of cells and assayed spectrophotometrically as described by Jakuboswski and colleagues^25^. Enzyme activity was calculated using an extinction coefficient of 43.66 M^−1^ cm^−1^ for H_2_O_2_ and catalase activity was expressed as µmol of H_2_O_2_/min- mg of protein (U/mg protein). For the oxidized and total glutathione, supernatant extracts were obtained from 100 mg of cells and were used for glutathione determination by the method of the glutathione reductase^58^. For the glutathione linked to proteins, pellet extracts were employed and glutathione was measured as described in Grant *et al*.^59^, but by adapting to our growth conditions. The amount of glutathione was expressed as nmol/mg of cells. Experiments were carried out in triplicate.

### Western blotting

The cells from the different growth conditions were taken and frozen to be broken afterward with one volume of glass beads in lysis buffer (Tris-HCl 0.1 M, pH 7.5, NaCl 0.5 M, MgCl_2_ 0.1 M, NP40 1% (v/v), PMSF 10 mM and protease inhibitors and phosphatase inhibitors) in a FastPrep 24 shaker for three cycles of 20 seconds^16^. Extracts were clarified by centrifugation and were diluted after quantification by the Bradford method (Biorad Inc. Hercules, CA, USA) in loading buffer for SDS-PAGE. After electrophoresis in an Invitrogen mini-gel device, the gel was blotted onto PVDF membranes for the Western blot analysis with a Novex semy dry blotter (Invitrogen, Carlsbad, CA, USA). The anti-phospho-S6 ribosomal protein (1:1000 dilution) and the anti-PRX antibodies (1:1000 dilution) were obtained from Cell Signalling Technology (Beverly, MA, USA). The anti-Rps6 antibody (1:1000 dilution) was acquired from Abcam (Cambridge, MA, USA), and anti-GFP (1:1000 dilution) and anti-HA (1:2000 dilution) were purchased from Santa Cruz Biotechnology (Santa Cruz, CA, USA). Secondary antibodies HRP-conjugated were purchased from Santa Cruz Biotechnology and used at a dilution of 1:5000. The ECL Western blotting detection system (GE) was used following the manufacturer’s instructions.

### Metabolomic analysis

Five cultures of C9 and C9 *trr1*Δ were grown in MS300 synthetic must, and were taken in the stationary phase when remaining sugars were around 50 g/l. Cell pellets were frozen and analyzed by Metabolon Inc. (Durham, NC, USA). Samples were prepared in the MicroLab STAR system (Hamilton Company). The resulting extract was divided into four fractions: two for the analysis by two separate reverse phase (RP)/UPLC-MS/MS methods with positive ion mode electrospray ionization (ESI), one for the analysis by RP/UPLC-MS/MS with the negative ion mode ESI, and one for the analysis by HILIC/UPLC-MS/MS with the negative ion mode ESI. A principal components analysis was performed. Each principal component is a linear combination of every metabolite, and principal components are uncorrelated. The number of principal components equals the number of observations.

## Electronic supplementary material

Supplementary Information

Supplementary Dataset 1

## Data Availability

All data generated or analysed during this study are included in this published article (and its Supplementary Information Files).
