# Supplementary material for: Yeast thioredoxin reductase Trr1p controls TORC1-regulated processes
Source: Sci Rep. 2018 Nov 7;8:16500. doi: 10.1038/s41598-018-34908-4 (PMC6220292; doi:10.1038/s41598-018-34908-4)
Supplement: Supplementary file 1 — Supplementary Information [file 41598_2018_34908_MOESM1_ESM.pdf]

Supplementary Information for the manuscript "Yeast thioredoxin reductase Trr1p controls TORC1-regulated processes" by Cecilia Picazo, Emilia Matallana[1] and Agustín Aranda, I2SysBio UV-CSIC.

This file contains the full images of the western blot analysis shown in Figures 2B and 6.

Supplementary Figure S1. The autophagy analysis under rapamycin treatment of the Pgk1-GFP fusion of the *trr1*Δ mutant. The arrow indicates the GFP fragment that marks autophagy.

Supplementary Figure S2 Full blot image for Figure 2B with cropped region marked with rectangle.

Supplementary Figure S3. Full blot images for Figure 6A with cropped regions marked with rectangles.

Supplementary Figure S4. Full blot images for Figure 6B with cropped regions marked with rectangles.

Supplementary Figure S5. Full blot image for Figure 6C with cropped region marked with rectangle.

Supplementary Figure S6. Full blot image for Supplementary Figure S1 with cropped region marked with rectangle.

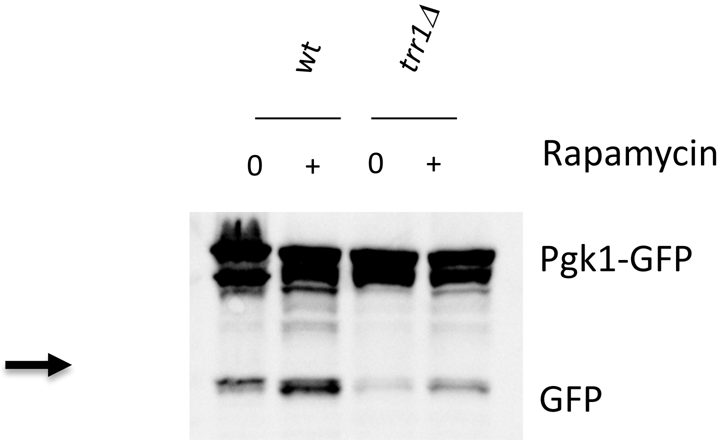

Supplementary Figure S1. The autophagy analysis under rapamycin treatment (200 nM rapamycin 30 minutes) of the Pgk1-GFP fusion of the *trr1*Δ mutant. The arrow indicates the GFP fragment that marks autophagy.

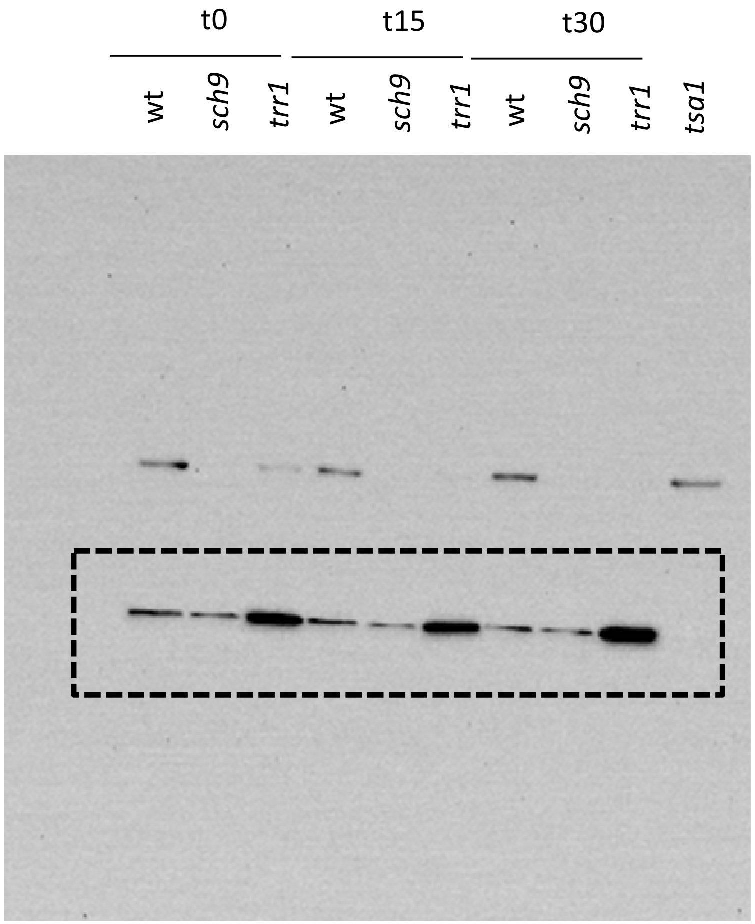

Peroxiredoxin antibody

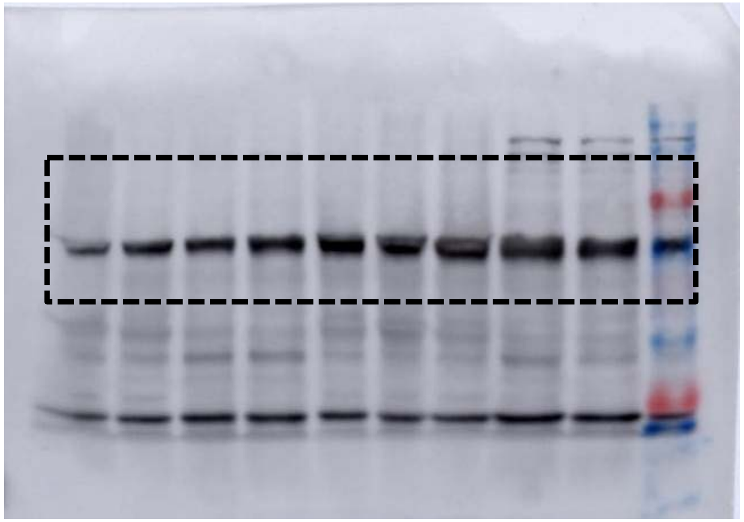

Tubulin antibody

Supplementary Figure S2. Full blot image for Figure 2B with cropped region marked with rectangle.

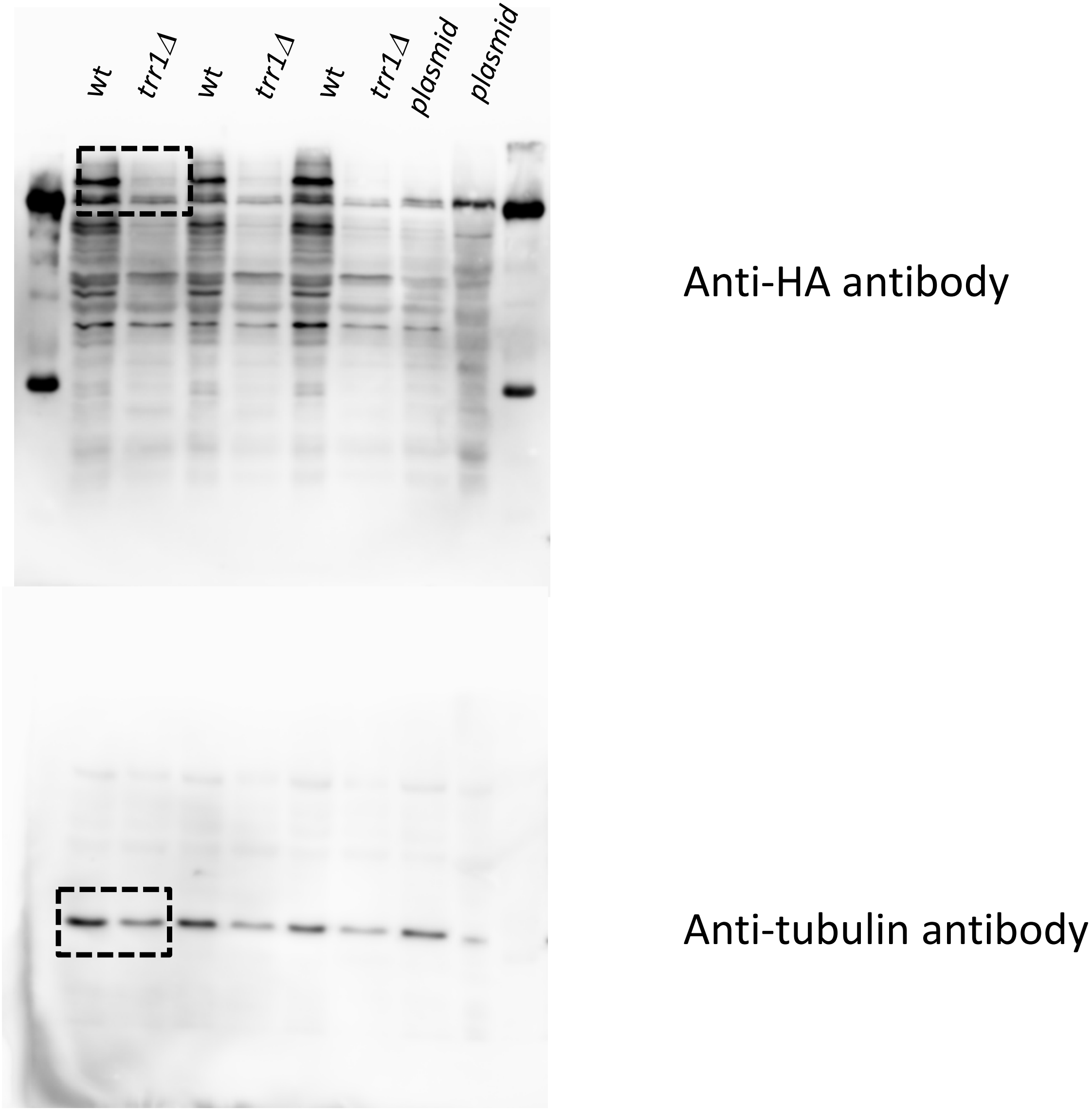

Supplementary Figure S3. Full blot images for Figure 6A with cropped regions marked with rectangles.

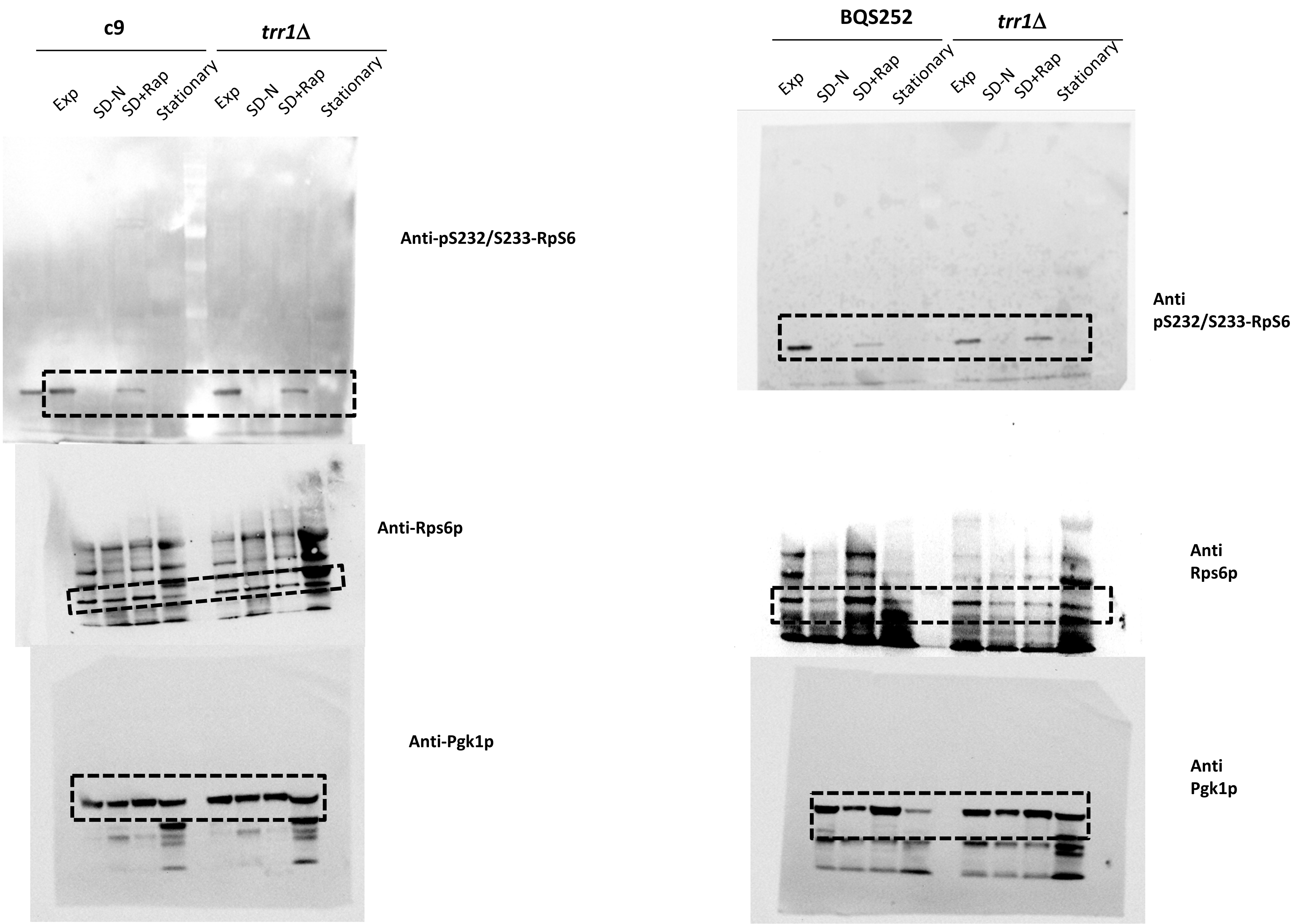

Supplementary Figure S4. Full blot images for Figure 6B with cropped regions marked with rectangles.

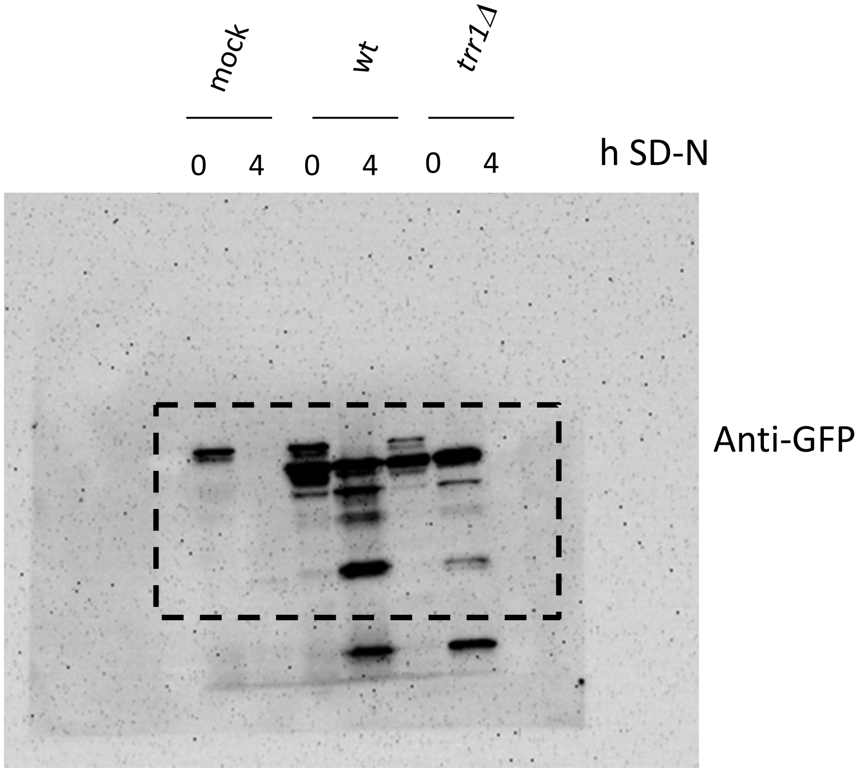

Supplementary Figure S5. Full blot images for Figure 6C with cropped region marked with rectangle.

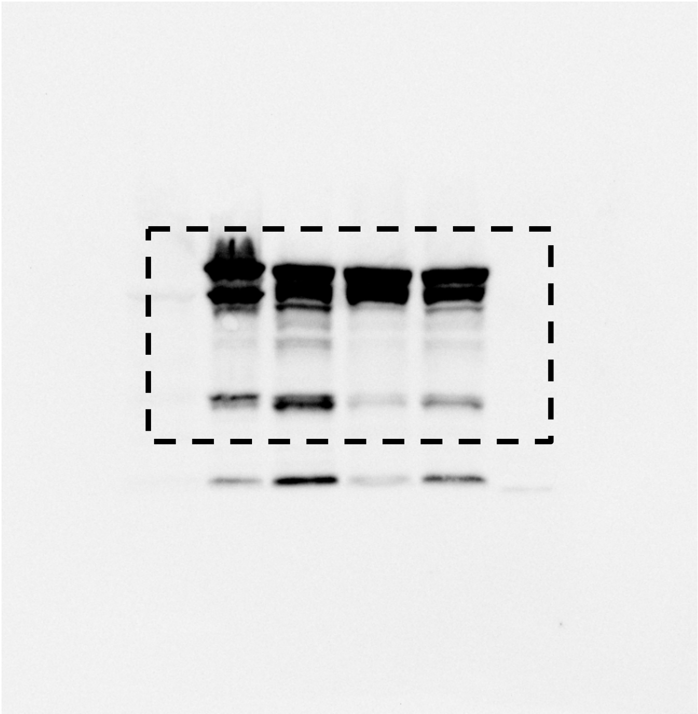

Supplementary Figure S6. Full blot image for Supplementary Figure S1 with cropped region marked with rectangle.
